# Supplementary material for: Effects of Different Exercises Combined with Different Dietary Interventions on Body Composition: A Systematic Review and Network Meta-Analysis
Source: Nutrients. 2024 Sep 5;16(17):3007. doi: 10.3390/nu16173007 (PMC11397086; doi:10.3390/nu16173007)
Supplement: Supplementary file 1 [file nutrients-16-03007-s001.zip › supplement.pdf]

Supplementary File S1. Searching Strategy
Supplementary Figures S1. Risk of bias summary
Supplementary Figures S2. Risk of bias graph
Supplementary Table S1. Characteristic of included trials.
Supplementary Table S2. Effects of aerobic exercise combined with different dietary interventions on body weight
Supplementary Table S3. Effects of resistance exercise combined with different dietary interventions on body weight
Supplementary Table S4. Effects of mixed exercise combined with different dietary interventions on body weight
Supplementary Table S5. Effects of aerobic exercise combined with different dietary interventions on body fat percentage
Supplementary Table S6. Effects of mixed exercise combined with different dietary interventions on body fat percentage
Supplementary Table S7. Effects of resistance exercise combined with different dietary interventions on lean body mass
Supplementary Table S8. Effects of mixed exercise combined with different dietary interventions on lean body mass

Supplementary File S1. Searching Strategy

PubMed

((("Exercise"[Mesh]) AND ((("Caloric Restriction"[Mesh]) OR ("Diet, Ketogenic"[Mesh])) OR ("Intermittent Fasting"[Mesh]))) AND ((((((((("Body Weight"[Mesh]) OR ("Body Mass Index"[Mesh])) OR ("Bone Density"[Mesh])) OR ("Blood Glucose"[Mesh])) OR ("Triglycerides"[Mesh])) OR ("Lipoproteins, HDL"[Mesh])) OR ("Lipoproteins, LDL"[Mesh])) OR ("Cholesterol"[Mesh])) OR ("Blood Pressure"[Mesh]))) AND ((randomized controlled trial[Publication Type] OR randomized[Title/Abstract] OR placebo[Title/Abstract]OR RCT[Title/Abstract]))

Web of Science

#1

TS=（Exercise）OR TS=（Exercises）OR TS=（Physical Activity）OR TS=（Activities, Physical）OR TS=（Activity, Physical）OR TS=（Physical Activities）OR TS=（Exercise, Physical）OR TS=（Exercises, Physical）OR TS=（Physical Exercise）OR TS=（Physical Exercises）OR TS=（Acute Exercise）OR TS=（Acute Exercises）OR TS=（Exercise, Acute）OR TS=（Exercises, Acute）OR TS=（Exercise, Isometric）OR TS=（Exercises, Isometric）OR TS=（Isometric Exercises）OR TS=（Isometric Exercise）OR TS=（Exercise, Aerobic）OR TS=（Aerobic Exercise）OR TS=（Aerobic Exercises）OR TS=（Exercises, Aerobic）OR TS=（Exercise Training）OR TS=（Exercise Trainings）OR TS=（Training, Exercise）OR TS=（Trainings, Exercise）

#2

TS=（Caloric Restriction ）OR TS=（Restriction, Caloric）OR TS=（Calorie Restricted Diet）OR TS=（Calorie Restricted Diets）OR TS=（Diet, Calorie Restricted）OR TS=（Restricted Diet, Calorie）OR TS=（Caloric Restricted）OR TS=（Restricted, Caloric）OR TS=（Low-Calorie Diet）OR TS=（Diet, Low-Calorie）OR TS=（Low Calorie Diet）OR TS=（Low-Calorie Diets）

#3

TS= (Diet, Ketogenic) OR TS= (Ketogenic Diet) OR TS= (Diets, Ketogenic) OR TS= (Ketogenic Diets）

#4

TS=（Intermittent Fasting）OR TS=（Fasting, Intermittent）OR TS=（Meal Skipping）OR TS=（Skipping, Meal）OR TS=（Breakfast Skipping）OR TS=（Skipping, Breakfast）OR TS=（Time Restricted Fasting）OR TS=（Fasting, Time Restricted）OR TS=（Restricted Fastings, Time）OR TS=（Time Restricted Eating）OR TS=（Eating, Time Restricted）OR TS=（Time Restricted Feeding）OR TS=（Feeding, Time Restricted）OR TS=（Time Restricted Feedings）

#5

TS=（Body Weight）OR TS=（Body Weights）OR TS=（Weight, Body）OR TS=（Weights, Body）

#6

TS=（Body Mass Index）OR TS=（Index, Body Mass）OR TS=（Quetelet Index）OR TS=（Index, Quetelet）OR TS=（Quetelet's Index）OR TS=（Quetelets Index）

#7

TS=（Bone Density）OR TS=（Bone Densities）OR TS=（Density, Bone）OR TS=（Bone Mineral Density）OR TS=（Bone Mineral Densities）OR TS=（Density, Bone Mineral）OR TS=（Bone Mineral Content）OR TS=（Bone Mineral Contents）

#8
TS=（Blood Glucose）OR TS=（Blood Sugar）OR TS=（Sugar, Blood）OR TS=（Glucose, Blood）
#9
TS= (Triglycerides) OR TS= (Triacylglycerols) OR TS= (Triacylglycerol) OR TS= (Triglyceride)
#10
TS=（Lipoproteins, HDL）OR TS=（HDL Lipoproteins）OR TS=（High-Density Lipoprotein）OR TS=（Lipoprotein, High-Density）OR TS=（High-Density Lipoproteins）OR TS=（High Density Lipoproteins）OR TS=（Lipoproteins, High-Density）OR TS=（alpha-Lipoproteins）OR TS=（alpha Lipoproteins）OR TS=（Heavy Lipoproteins）OR TS=（Lipoproteins, Heavy）OR TS= (High Density Lipoprotein) OR TS= (Density Lipoprotein, High) OR TS= (Lipoprotein, High Density）OR TS=（alpha-Lipoprotein）OR TS=（alpha Lipoprotein）OR TS=（alpha-1 Lipoprotein）
#11
TS=（Lipoproteins, LDL）OR TS=（LDL Lipoproteins）OR TS=（beta-Lipoprotein）OR TS= （beta Lipoprotein) OR TS= (Low-Density Lipoproteins) OR TS= (Lipoproteins, Low-Density) OR TS= (Low Density Lipoproteins) OR TS= (beta-Lipoproteins) OR TS= (beta Lipoproteins) OR TS= (Low-Density Lipoprotein) OR TS= (Lipoprotein, Low-Density) OR TS= (Low Density Lipoprotein）OR TS=（LDL-2）OR TS=（LDL2）OR TS=（Low-Density Lipoprotein 2）OR TS=（Low Density Lipoprotein 2）OR TS=（LDL(2)）OR TS=（LDL-1）OR TS=（LDL1）OR TS=（Low-Density Lipoprotein 1）OR TS=（Low Density Lipoprotein 1）OR TS=（LDL(1)）
#12
TS=（Cholesterol）OR TS=（Epicholesterol）
#13
TS= (Blood Pressure) OR TS= (Pressure, Blood) OR TS= (Diastolic Pressure) OR TS= (Pressure, Diastolic）OR TS=（Pulse Pressure）OR TS=（Pressure, Pulse）OR TS=（Systolic Pressure）OR TS=（Pressure, Systolic）OR TS=（Pressures, Systolic）
#14
#2 OR #3 OR #4
#15
#5 OR #6 OR #7 OR #8 OR #9 OR #10 OR #11 OR #12 OR #13
#16
TS=（randomized controlled trial）OR TS=（randomized）OR TS=（placebo）
#17
#1 AND #14 AND #15 AND #16

Embase
#1
'exercise'/exp
#2
'caloric restriction'/exp OR 'ketogenic diet'/exp OR 'intermittent fasting'/exp
#3

'body weight'/exp OR 'body mass'/exp OR 'bone density'/exp OR 'glucose blood level'/exp OR 'triacylglycerol'/exp OR 'high density lipoprotein'/exp OR 'low density lipoprotein'/exp OR 'cholesterol'/exp OR 'blood pressure'/exp
#4
'randomized controlled trial'/exp
#5
#1 AND #2 AND #3 AND #4

Cochrane Library
#1 MeSH descriptor: [Exercise] explode all trees
#2 MeSH descriptor: [Caloric Restriction] explode all trees
#3 MeSH descriptor: [Diet, Ketogenic] explode all trees
#4 MeSH descriptor: [Intermittent Fasting] explode all trees
#5 MeSH descriptor: [Body Weight] explode all trees
#6 MeSH descriptor: [Body Mass Index] explode all trees
#7 MeSH descriptor: [Bone Density] explode all trees
#8 MeSH descriptor: [Blood Glucose] explode all trees
#9 MeSH descriptor: [Triglycerides] explode all trees
#10 MeSH descriptor: [Lipoproteins, HDL] explode all trees
#11 MeSH descriptor: [Lipoproteins, LDL] explode all trees
#12 MeSH descriptor: [Cholesterol] explode all trees
#13 MeSH descriptor: [Blood Pressure] explode all trees
#14 #2 OR #3 OR #4
#15 #5 OR #6 OR #7 OR #8 OR #9 OR #10 OR #11 OR #12 OR #13
#16 #1 AND #14 AND #15

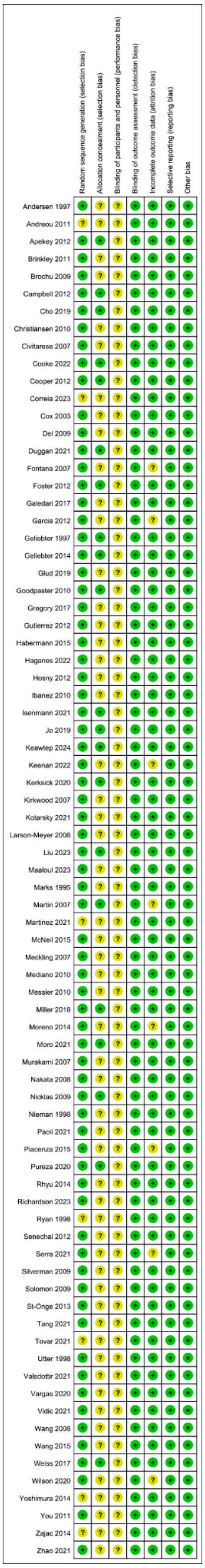

Figure S1. Risk of bias summary

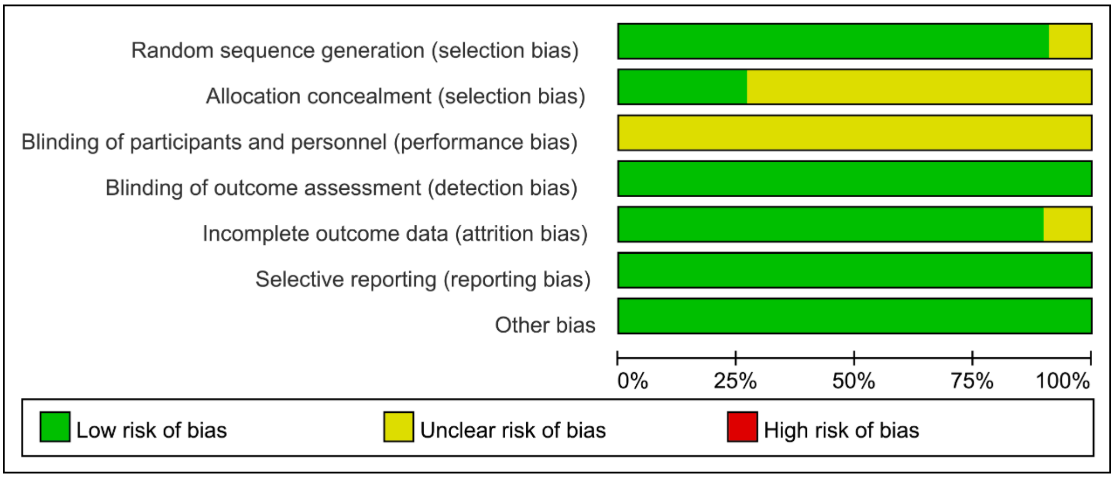

Figure S2. Risk of bias graph

Table S1. Characteristic of included trials.

	Study	Sample Characteristics	Type of intervention	Duration	main indicators
1	Andersen 1997[93]	Healthy obese women，38 years old	CR, CR+EX	24w	BW, FM, FFM,
2	Andreou 2011[94]	Healthy obese women，33 years old	CR, CR+EX	18w	BW, BMI, FM%,
3	Apekey 2012[95]	Obese adults, 18 years old	CR, CR+EX	8w	BW, BMI, TG, HDL, LDL, TC, SBP, DBP
4	Brinkley 2011[96]	Overweight and obese postmenopausal women, 58 years old	CR, CR+EX	20w	BW, BMI, FM%, FM, FFM, TG, HDL, LDL, TC, SBP, DBP
5	Brochu 2009[97]	Overweight and obese postmenopausal women, 58 years old	CR, CR+EX	6m	BW, BMI, TG, HDL, LDL, TC, SBP, DBP
6	Campbell 2012[98]	Healthy obese women, 60 years old	CON, CR, EX, CR+EX	12m	BW, FM%,
7	Cho 2019[99]	Overweight or obese adults, 35 years old	ADF+EX, ADF, EX, CON	8w	BW, BMI, FM%, FM, TG, HDL, LDL, TC,
8	Christiansen 2010[100]	Obese men and women, 34	EX, CR, CR+EX	12w	BW, BMI,
9	Civitarese 2007[101]	healthy person, 37 years old	CON, CR, CR+EX	6m	BW, FM%, FM, FFM,
10	Cooke 2022[10	Overweight and obese individuals, 35.4 years old	EX, 5/2F, 5/2F+EX	16w	BW, FM, FFM, TG,

	2]				HDL, LDL, TC, SBP, DBP
11	Cooper 2012[103]	Severely obese adults, 33 years old	CR, CR+EX	6m	BW, BMI, FM, FFM, HDL, LDL, TC, SBP, DBP
12	Correia 2023[104]	Trained sports student, 23.7 years old	TRF16/8+EX, EX	4w	BW, FM, FFM,
13	Cox 2003[105]	Overweight men, 42 years old	EX, CR+EX	16w	BW, FM, FFM,
14	Del 2009[106]	Healthy overweight premenopausal women, 34 years old	CR+EX, CR	22w	BW, BMI, FM%, FM, FFM,
15	Duggan 2021[107]	Overweight healthy postmenopausal women, 62 years old	CON, CR, EX, CR+EX	12m	BMI, FM%,
16	Fontana 2007[108]	Non-obese subjects, 57 years old	EX, CR, CON	12m	TG, HDL, LDL, TC, SBP, DBP
17	Foster 2012[109]	Overweight to obese postmenopausal women, 58 years old	CON, CR, EX, CR+EX	12m	BW, BMI, FM%, FM, FFM,
18	Galedari 2017[110]	Untrained overweight male, 30 years old	CR, CR+EX	12w	BW, FM, FFM,
19	Garcia 2012[111]	Obese women, 50 years old	CON, CR, CR+EX	16w	BW, TG, HDL, TC,
20	Geliebter 1997[112]	Moderately obese people, 30 years old	CR+EX, CR	8w	BW, FM, FFM,
21	Geliebter 2014[113]	Overweight and obese people, 35 years old	CR+EX, CR	8w	BW, BMI, FM, FFM, TG, HDL, LDL, SBP, DBP
22	Glud 2019[114]	Overweight and obese healthy people, 36 years old	EX, CR, CR+EX	12w	BW, BMI,

23	Goodpaster 2010[115]	Severely obese adults, 46 years old	CR+EX, CR	6m	BW, BMI, FM, FFM, TG, HDL, TC, SBP, DBP
24	Gregory 2017[116]	Non-elite CrossFit subjects, 35 years old	EX, KD+EX	6w	BW, BMI, FM%, FM,
25	Gutierrez 2012[117]	Obese subjects, 42 years old	CR, CR+EX	3m	BW, BMI, FM%,
26	Hadizadeh 2020[118]	No training experience, 35 years old	KD+EX, EX	12m	BW, BMI, FM, FFM,
27	Haganes 2022[119]	Overweight to obese women, 36 years old	CON, TRF, EX, TRF+EX	7w	BW, FM, TG, HDL, LDL, TC, SBP, DBP
28	Hosny 2012[120]	Obese premenopausal women, 35 years old	CR, CR+EX	3m	BW, BMI, FFM,
29	Ibanez 2010[121]	Obese women, 50 years old	CON, CR, CR+EX	16w	BW, BMI, FFM,
30	Isenmann 2021[122]	Fitness enthusiasts with a BMI greater than 25, 27 years old	TRF+EX, EX	14w	BW, BMI, FM, FFM,
31	Jo 2019[123]	Obese people, 58 years old	CR, CR+EX	12w	BW, BMI, FM%, FM, FFM,
32	Keawtep 2024[124]	Obese postmenopausal women, 53 years old	CON, 5/2F, EX, 5/2F+EX	3m	BW, BMI, FM%, FM, FFM, TG, TC,
33	Keenan 2022[125]	Healthy men and women, 24 years old	5/2F+EX, CR+EX	12w	TG, HDL, LDL, TC,
34	Kerksick 2020[126]	Postmenopausal women, 51 years old	EX, CR+EX	14w	BW, FM%, FM, FFM, TG, HDL, LDL, TC,

					SBP, DBP
35	Kirkwood 2007[127]	Overweight women, 41 years old	CON, CR, EX, CR+EX	12w	BW, BMI, FM%,
36	Kotarsky 2021[128]	Overweight and obese adults, 44 years old	EX, TRF+EX	8w	BW, BMI, FM, FFM, HDL, TC,
37	Larson-Meyer 2008[129]	Overweight and obese adults, 40 years old	CR, CR+EX, CON	6m	BW, FM%,
38	Liu 2023[130]	Female college students suffering from hidden obesity, 20 years old	CON, TRF, EX, TRF+EX	8w	BW, BMI, FM%, FM, FFM, TG, HDL, LDL, TC, SBP, DBP
39	Maaloul 2023[131]	Obese men, 32 years old	TRF, TRF+EX	1m	BW, FM%, FM, FFM, TG, HDL, LDL, TC,
40	Marks 1995[132]	Overweight, inactive women, 38 years old	CON, CR, CR+EX	20w	BW, FM%, FM, FFM,
41	Martin 2007[133]	Overweight people, 37 years old	CON, CR, CR+EX	6m	FM, FFM,
42	Martinez 2021[134]	Active, normal-weight women, 27 years old	EX, TRF+EX	8w	BW, FM%, FM, FFM,
43	McNeil 2015[135]	Overweight/obese postmenopausal women, 58 years old	CR, CR+EX	6m	BW, FM, FFM,
44	Meckling 2007[136]	Overweight/obese women, 45 years old	CR, CR+EX	12w	BW, BMI, FM%, FM, FFM,
45	Mediano 2010[137]	Non-obese women, 37 years old	CR, CR+EX	12M	BW, BMI, FM%, TG, HDL, LDL, TC,
46	Messier	Overweight and obese	CR, CR+EX	6M	BW, BMI,

	2010[138]	postmenopausal women, 57 years old			FM, FFM,
47	Miller 2018[139]	Premenopausal women, 32 years old	CON, CR, EX, CR+EX	4m	BW, FM%, FM, FFM,
48	Moreno 2014[140]	Obese patients, 45 years old	KD, CR	12m	TG, HDL, LDL, TC,
49	Moro 2021[141]	Endurance training experience, 30 years old	TRF+EX, EX	12m	BW, FM, FFM, TG, HDL, LDL, TC,
50	Murakami 2007[142]	Obese non-diabetic patients, 50 years old	CR, CR+EX	12w	BW, BMI, FM, TG, HDL, LDL, TC, SBP, DBP
51	Nakata 2008[143]	Overweight premenopausal Japanese women, 41 years old	CR, CR+EX	14w	BW, FM, FFM,
52	Nicklas 2009[144]	Overweight and obese postmenopausal women, 58 years old	CR, CR+EX	20w	BW, FM%, FM, FFM,
53	Nieman 1998[145]	Obese women, 45 years old	CON, EX, CR, CR+EX	12w	BW, BMI, FM%, TG, TC,
54	Paoli 2021[146]	Male bodybuilder, 27 years old	KD+EX, EX	8w	BW, FM, FFM, TG, HDL, LDL, TC,
55	Piacenza 2015[147]	Overweight adult subjects, 37 years old	CON, CR, CR+EX	6m	BW, BMI,
56	Pureza 2020[148]	Obese women, 30 years old	CR, TRF	3w	BW, BMI, FM%, SBP, DBP
57	Rhyu 2014[149]	Ordinary students, 17 years old	KD+EX, EX	3w	BW, BMI, FM%, FFM,
58	Richardson 2023[150]	Male runner, 29 years old	EX, TRF+EX	4w	BW, FM%, FM, FFM, TG, HDL, LDL, TC,

					SBP, DBP
59	Ryan 1998[151]	Older women, 63 years old	CR, CR+EX	6m	BW, BMI, FM%, FM, FFM,
60	Senechal 2012[152]	Postmenopausal women, 62 years old	CON, CR, EX, CR+EX	12w	BW, FM%, FM, FFM,
61	Serra 2021[153]	Overweight, postmenopausal women, 60 years old	CR, CR+EX	6m	BW, BMI,
62	Silverman 2009[154]	Overweight postmenopausal women, 60 years old	CR, CR+EX	6m	BW, BMI, FM, FFM,
63	Solomon 2009[155]	Obese elderly people, 66 years old	EX, CR+EX	12w	BW, BMI, FM, FFM, TG, TC,
64	St-Onge 2013[156]	Overweight or obese postmenopausal women, 58.3 years old	CR, CR+EX	6m	BW, FM, FFM,
65	Tang 2021[157]	Bachelor student, 20 years old	CR, EX, CR+EX	8w	BW, BMI, FM%, FM, TG, HDL, LDL, TC, SBP, DBP
66	Tovar 2021[158]	Male runner doing endurance training, 29 years old	EX, TRF+EX	4w	BW, FM%, FM, FFM,
67	Utter 1998[159]	Obese women, 45 years old	CON, EX, CR, CR+EX	12w	BW, BMI, FM%, FM, FFM,
68	Valsdottir 2021[160]	Overweight and obese women, 40 years old	CON, KD, EX, KD+EX	10w	BW, FM%, FM, FFM, TG, HDL, LDL, TC,
69	Vargas 2020[161]	Trained women, 28 years old	KD+EX, EX	8w	BW, FM, FFM,
70	Vidic 2021[162]	Middle-aged men with resistance training experience, 42 years old	KD+EX, EX	4w	BW, FM, FFM, TG, HDL, LDL,

					TC,
71	Wang 2008[163]	Overweight and obese women, 59 years old	CR, CR+EX	20w	BW, FM%, FM, FFM,
72	Wang 2015[164]	Overweight or obese postmenopausal women, 58 years old	CR, CR+EX	20w	BW, BMI, FM%, FM, FFM,
73	Weiss 2017[165]	Overweight, sedentary women and men, 57 years old	CR, EX, CR+EX	17w	BW, FM, FFM, SBP, DBP
74	Wilson 2020[166]	Resistance Training for Men, 22 years old	KD+EX, EX	10w	BW, TG, HDL, TC,
75	Yoshimura 2014[167]	Adults with visceral obesity, 55 years old	CR, CR+EX	12w	BW, BMI, FM%, FM, FFM, TG, HDL, LDL, TC, SBP, DBP
76	You 2011[168]	Obese women, 59 years old	CR, CR+EX	20w	BW, FM%, FM,
77	Zajac 2014[169]	Mountain bike rider, 28 years old	EX, KD+EX	4w	BW, BMI, FM%,
78	Zhao 2021[170]	Overweight or obese men, 49.5 years old	CON, CR+EX	12w	BW, BMI, FM%,

CON=The group without any intervention， EX=Exercise， CR=Caloric Restriction， 5/2F=5/2intermittent fasting， TRF=Time-restricted Fasting， KD=Ketogenic Diet。BW=Body Weight, BMI=Body Mass Index, FM%=Fat Percentage, FM=Fat Mass, FFM=Fat Free Mass, TG= Triglycerides, HDL= High-density Lipoprotein, LDL= Low-density Lipoprotein, TC= Total Cholesterol, SBP= Systolic Blood Pressure, DBP= Diastolic Blood Pressure.

Table S2. Effects of aerobic exercise combined with different dietary interventions on body weight

Treatm~t	SUCRA	PrBest	Mean Rank
CR+EX	85.2	21.5	2.3
5/2F+EX	39.7	3.9	6.4
TRF+EX	47.7	1.5	5.7
KD+EX	51.7	4.7	5.3

Table S3. Effects of resistance exercise combined with different dietary interventions on body weight

Treatm~t	SUCRA	PrBest	Mean Rank
CR+EX	57.1	7.3	3.1
TRF+EX	81.6	54.5	1.9
KD+EX	76.3	33.3	2.2

Table S4. Effects of mixed exercise combined with different dietary interventions on body weight

Treatm~t	SUCRA	PrBest	Mean Rank
CR+EX	49.9	8.6	5
5/2F+EX	67.8	11	3.6
TRF+EX	44.7	1.7	5.4
KD+EX	29.5	3.2	6.6

Table S5. Effects of aerobic exercise combined with different dietary interventions on body fat percentage

Treatm~t	SUCRA	PrBest	Mean Rank
CR+EX	87.4	36.8	1.9
TRF+EX	22.1	0	6.5
KD+EX	24.9	0.2	6.3

Table S6. Effects of mixed exercise combined with different dietary interventions on body fat percentage

Treatm~t	SUCRA	PrBest	Mean Rank
CR+EX	79.9	40.8	2.6
5/2F+EX	45.1	2.9	5.4
TRF+EX	57.9	15.4	4.4
KD+EX	34.7	4.9	6.2

Table S7. Effects of resistance exercise combined with different dietary interventions on lean body mass

Treatm~t	SUCRA	PrBest	Mean Rank
CR+EX	57.3	9.9	3.1
TRF+EX	34.6	9.6	4.3
KD+EX	16.6	1.4	5.2

Table S8. Effects of mixed exercise combined with different dietary interventions on lean body mass

Treatm~t	SUCRA	PrBest	Mean Rank
CR+EX	75.5	32.2	2.7
5/2F+EX	14.1	0.1	7
TRF+EX	76.4	32.6	2.7
